# A unified framework for link prediction based on non-negative matrix factorization with coupling multivariate information

**DOI:** 10.1371/journal.pone.0208185

**Published:** 2018-11-29

**Authors:** Wenjun Wang, Minghu Tang, Pengfei Jiao

**Affiliations:** 1 School of Computer Science and Technology, College of Intelligence and Computing, Tianjin University, Tianjin, China; 2 School of Computer Science and Technology, Qinghai Nationalities University, Qinghai, China; Liverpool John Moores University, UNITED KINGDOM

## Abstract

Many link prediction methods have been developed to infer unobserved links or predict missing links based on the observed network structure that is always incomplete and subject to interfering noise. Thus, the performance of existing methods is usually limited in that their computation depends only on input graph structures, and they do not consider external information. The effects of social influence and homophily suggest that both network structure and node attribute information should help to resolve the task of link prediction. This work proposes SASNMF, a link prediction unified framework based on non-negative matrix factorization that considers not only graph structure but also the internal and external auxiliary information, which refers to both the node attributes and the structural latent feature information extracted from the network. Furthermore, three different combinations of internal and external information are proposed and input into the framework to solve the link prediction problem. Extensive experimental results on thirteen real networks, five node attribute networks and eight non-attribute networks show that the proposed framework has competitive performance compared with benchmark methods and state-of-the-art methods, indicating the superiority of the presented algorithm.

## Introduction

As a very important research direction in complex networks, link prediction is attracting a large number of researchers from different disciplines, including computer science, biology, physics and sociology, because of its wide application. It aims to infer the likelihood of the existence of a link between two nodes unconnected by means of the known structure information in the network [1–3]. Link prediction can be used to explore the evolution mechanism of the network [4,5], recommend trusted partners in business trade [6], recommend travel hotspots [7,8], mine suspects in counterterrorism networks [9–11], analyse criminal networks [12,13] and so on.

In recent years, with the development of complex network research, people have proposed many ways to predict the links for specific networks in different fields from various perspectives [14–16]. In simple terms, the existing methods for link prediction can be divided into three categories: unsupervised, supervised and other mixed methods. i) The first computes similarity scores between two nodes based on the known topological structure of the network. It is one of the most widely used methods in recent years and methods such as Common neighbour(CN), Adamic-Adar index(AA), and Resource Allocation index(RA), became the baseline for judging new methods [1]. This kind of method only depends on the information of known topology structure in network. Therefore, its prediction results are easily affected by network data sparsity (The number of edges known to be present is often significantly less than the number of edges known to be absent.). In fact, this is still the biggest challenge in the current research of link prediction. ii) The supervised approaches, on the other hand, attempt to be directly predictive of link behaviour. They generally need to find the characteristics of the node interaction and learn latent features from the topological structure of network [17–19]. Our work is to use this method to achieve multiple attribute fusion techniques to improve prediction performance. iii) The mixed methods include many methods, such as those mainly based on the probability model, perturbation-based frameworks, and matrix completion, etc. The probability model is inherently high cost in computational complexity since its application is limited [20,21]. In addition, structural perturbation-based and matrix completion methods are the most recently proposed the state-of-the-art approaches. Lü LY et al. [22] assumed that the regularity of a network is reflected in the consistency of structural features before and after a random removal of a small set of links. Based on the perturbation of the adjacency matrix, they proposed a universal structural consistency index that is free of prior knowledge of the network organisation. Furthermore, Xu XY [23] and Wang WJ et al. [24] proposed a perturbation framework based on matrix decomposition for link prediction. On the other hand, Pech Ratha et al. [25] proposed a method for link prediction based on matrix completion.

Although these methods can achieve prediction tasks, there is still a shortcomings of insufficient useful information to some extent. Moreover, they are always challenged by high computational costs and data sparsity and network noise. In addition, with the increase of data scale, how the proposed method can be scalable, transplantable and robust in large-scale networks becomes the evaluation basis of the algorithm. Therefore, how to mine the network features, solve the above challenges and improve the performance of link prediction become the main concerns in this paper.

In fact, a complex network is an abstraction of real world, where the nodes represent entities that have very rich attribute information in the real environment. For example, individuals in online social networks have sociological characteristics such as gender, age, religious belief, educational background, and hobbies. The principle of social influence and homophily show that users with similar attributes, or in some cases antithetical attributes, are likely to link to one another [26–28], motivating the use of attribute information for link prediction. Additionally, some previous studies have also empirically demonstrated that non-topological information such as node attributes has a certain impact on the formation and evolution of social networks [29–32]. Therefore, network structure and node attribute information can be considered when predicting links.

In recent years, with the development of other fields related to complex networks, some methods of link prediction have been proposed based on the attribute information of nodes [33,34]. These methods, such as relational learning[35–37], semantic mining[16,33,38]. random walk[39,40], matrix factorization[41], have been proposed to leverage attribute information for link prediction. However, due to the diversity and heterogeneity of information and the difference of fusion methods, the overall effect of these algorithms is insufficient. Therefore, the algorithmic question of how to simultaneously incorporate these two sources of information remains largely unanswered. More recently, Gong N Z et al.[39] proposed an approach based on random walk algorithm to predict links as well as to infer node attributes, it suffers from scalability issues. Backstrom and Leskovec [42] presented a supervised random walk algorithm for link prediction, but this approach only incorporates node information for neighboring nodes. Taking these influence into account, we would like to consider: Can this external information about the nodes contribute to infer an interaction relationship between the nodes? What is the role of this external auxiliary information in predicting the interaction of nodes? How much dependency exists between external information and internal interaction? What methods of fusion are the most effective?

Because non-negative matrix factorization (NMF) [43, 44] has the advantages of non-negative, extensibility and interpretability of physical phenomena, it has been widely used in the study of complex networks [45–47]. For example, Yang et al. [48] designed a probabilistic latent variable model which combined the NMF and block structure of matrices for link prediction, but they did not use the node attribute information. Chen BL et al. [41] proposed a non-negative matrix factorization for link prediction that combines network structure and node-attribute information, but this approach does not fully explore the combination form of structure and attribute information in depth, and the complexity is high. As previous studies have shown that node sociological information can assist prediction, and NMF based on matrix decomposition not only has non-negative and interpretable advantages, but also can easily integrate heterogeneous information, make multiple information work together. Inspired by the advantages of non-negative matrix factorization, in this work, we use it to fuse heterogeneous multi-source information for link prediction problem.

In this paper, we propose a unified framework, SASNMF, for link prediction of coupled multivariate information based on NMF. The framework combines local information of a node attribute with global information of the topological structure to solve the link prediction problem from a new perspective of the macro/micro-level. Furthermore, the effects of different combinations of multivariate information on the prediction results are verified under the same framework. Experimental results on 13 real-world network datasets display that the proposed framework has competitive performance compared with baseline and several state-of-the-art algorithms, indicating the superiority of our algorithm. Specifically, this paper makes the following contributions.

First, we develop a prediction framework based on NMF, and auxiliary information from two different levels of macroscopic and microscopic information is coupled to realize the purpose of node relationship prediction.

Second, two kinds of auxiliary information are mined and used to alleviate the problem that the structural information cannot be fully utilized due to data sparsity and reduce the effect of the noise in the forecast.

Third, several different combination modes of auxiliary information are proposed, and the performance is compared and analysed separately under the same framework for the datasets with and without attributes.

## Materials and methods

### Preliminaries

In this section, we first describe the problem of link prediction. In addition, we review the conventional NMF method.

#### Problem description

For a social network can be represented as an undirected graph G = (V,E), where V = {*v*_1_,*v*_2_,⋯*v*_*n*_} is the set of users (nodes) and E ⊆ V × V is the set of existing relations (edges) between users. The interaction relation between nodes is formally marked as an adjacency matrix *A*_*n*×*n*_ in network with n vertices. The element of the i^th^ row and the j^th^ column in the matrix correspond to the link between node i and j in the network, where *A*_*ij*_ = 1 if there is a link from i to j and *A*_*ij*_ = 0 otherwise. Generally, the adjacency matrix A represents the macro-relations of the network topology. The problem of link prediction is inferring the probability of an existent link between nodes x and y based on known information in the network, and the probability is expressed as score *P*_*xy*_. The score can be viewed as the similarity of nodes x and y. The higher *P*_*xy*_ is, the more similar x is to y. According to the score, all nonexistent links in the network can be sorted in descending order. The links at the top are the most likely to exist. In this paper, we compute the score *P*_*xy*_ based on NMF.

To test the algorithm’s accuracy, the observed links, E, are randomly divided into two parts: the training set, E^train^ is treated as known information, while the probe set, E^test^ has no known information and is used for testing in the prediction experiment. The proportion of links in these two parts ranges from 90% to 20%. Thus, when the training set consists of 90% of links, the remaining 10% of links constitute the test set. Furthermore, in the experiment, we conducted the simulations of SASNMF 100 times for each network and only report the average values in this paper.

#### NMF review

Given a matrix $V\in R_{+}^{n\times m}$, the NMF aims to find two nonnegative factor matrices $W\in R_{+}^{n\times k}$ and $H\in R_{+}^{k\times m}$ that make V ≈ V′ = WH. In general, the k, (m + n)k ≪ mn, is the number of latent features or the inner rank of V. The matrix W is called the basis matrix, and H is the coefficient matrix. The column vector of the original matrix V is the weighted sum of all column vectors of matrix W, while the weighted coefficient is just the elements of the corresponding column vector of matrix H.

The optimization problem of NMF is a convex optimization problem[49]. Due to its NP-hardness and lack of appropriate convex formulations, the nonconvex formulations with relatively easy solvability are generally adopted, and only local minima are achievable in a reasonable computational time. Hence, the classic and also more practical approach is to perform alternating minimization of a suitable cost function as the similarity measures between V and the product WH[44].In this paper, our goal is to find V′ as an approximation of V to implement the task of link prediction. Then, the problem of link prediction in networks can be cast as the following NMF problem:

$$\min_{W\geq 0,H\geq 0\;}l(V,WH),$$ (1)

where l(,) is a general loss function. Generally speaking, the form of Euclidean distances are commonly used as this function. Assuming that there are two matrices X and Y, according to the definition of Euclidean distance, this loss function can be written as following form:

$$l(X,Y)={\left\|X-Y\right\|}_{F}^{2}=\sum_{ij}{\left|\left(X_{ij}-Y_{ij}\right)\right|}^{2}$$ (2)

In this work, we will also make use of such Euclidean loss. Then, our problem of link prediction is to solve the following optimization problem:

$$\min_{W,H}{\left\|V-WH\right\|}_{F}^{2}\;s.t.\;W\geq 0,H\geq 0$$ (3)

where ‖∙‖_*F*_ indicates the Frobenius norm, constrain W ≥ 0,H ≥ 0 requires that all the elements in matrices W and H are non-negative. The Frobenius norm of the matrix X is denoted by ${\left\|X\right\|}_{F}=\sqrt{\sum_{ij}{\left|x_{ij}\right|}^{2}}=\sqrt{tr(X^{H}X)}$.

Although there have been some notable results on NMF, they are far to be perfect with lots of open questions remained to be solved. More details can be found in Ref. 44.

## Methods

### Prediction framework: SASNMF

Because of the influence of the data sparsity, and that the observed links are only a small proportion of all possible links, the methods that rely solely on network structural information have the problem of low prediction accuracy. According to the introduction above, the influence of data sparsity can be alleviated, and the link prediction accuracy can be improved by using the auxiliary information of the network. Therefore, in this paper, we attempt to fully integrate the auxiliary information to make up for the incomplete topology information so that the prediction performance is improved. According to the NMF algorithm, we use the adjacent matrix *A*_*n*×*n*_, which represents the macroscopic information of the network topology structure, and the auxiliary attribute similarity matrix *S*_*n*×*n*_, which represents the microcosmic information, to create the NMF framework. Here, we need to find two nonnegative factors matrices W and H to satisfy the form of V ≈ WH. Thus, the matrix A is decomposed into $A=W_{1}H_{1},W_{1}\in R_{+}^{n\times k},H_{1}\in R_{+}^{k\times n}$, where k ≪ n. In the same way, the similarity matrix S is decomposed into $S=W_{2}H_{2},W_{2}\in R_{+}^{n\times m},H_{2}\in R_{+}^{m\times n}$, where m ≪ n. Then, we map these two pieces of information into two low-rank approximation spaces, in which *W*_1_ and *W*_2_ represent the bases in their latent spaces. According to formula (3), we have

$$\min_{W_{1},H_{1}}{\left\|A-W_{1}H_{1}\right\|}_{F}^{2}\;s.t.\;W_{1}\geq 0,H_{1}\geq 0$$ (4)

$$\min_{W_{2},H_{2}}{\left\|S-W_{2}H_{2}\right\|}_{F}^{2}\;s.t.\;W_{2}\geq 0,H_{2}\geq 0$$ (5)

However, our goal is to develop an indicator that can couple multivariate information to help improve the accuracy of link prediction. Therefore, formula (4) and (5) are combined into the following new form

$$Q=\min_{W_{1},H_{1}}{\left\|A-W_{1}H_{1}\right\|}_{F}^{2}+\min_{W_{2},H_{2}}{\left\|S-W_{2}H_{2}\right\|}_{F}^{2}$$ (6)

The information shown in the above formula (6) are only a simple combination of both the topological structure and auxiliary attribute, and they are not fully integrated into the same feature space. Therefore, we need to find a common factor matrix W to combine this information and then to make it a guider within the processing of the link prediction problem. That is, we develop a framework for link prediction that can employ a low-rank latent feature space representation to realize network structure prediction and add the lack of information within the network. Furthermore, let W = *W*_1_ = *W*_2_ to indicate that the two pieces of information in the network are mapped to the same feature space. At the same time, to avoid overfitting and to leverage the effects extent between the topology information and auxiliary attribute information in the link prediction results, we need to constrain and mediate the framework through setting up parameters. Finally, the objective function is created as follows:

$$Q=\min_{{W,H}_{1},H_{2}}({\left\|A-WH_{1}\right\|}_{F}^{2}+\alpha {\left\|S-WH_{2}\right\|}_{F}^{2}+\beta ({\left\|H_{1}\right\|}_{F}^{2}+{\left\|H_{2}\right\|}_{F}^{2}))$$ (7)

$$s.t.\;W\geq 0,H_{1}\geq 0,H_{2}\geq 0$$

where $W\in R_{+}^{n\times k},H_{1},H_{2}\in R_{+}^{k\times n}$, α is an equilibrium parameter for mediating the effect of the structure and attribute, and β is a regularization parameter to avoid overfitting.

Although it is difficult to obtain the global optimal solution of Q, the local can be implemented by a multiplicative iteration method.

To (7) decompose, by introducing the Lagrangian multiplier ψ,φ,ϕ for the nonnegativity of W, *H*_1_ and *H*_2_; we obtain the loss function without constraints:

$$L=\frac{1}{2}(({\left\|A-WH_{1}\right\|}_{F}^{2}+\alpha {\left\|S-WH_{2}\right\|}_{F}^{2}+\beta ({\left\|H_{1}\right\|}_{F}^{2}+{\left\|H_{2}\right\|}_{F}^{2}))+Tr(\psi^{T}W)+Tr(\varphi^{T}H_{1})+Tr(\phi^{T}H_{2})$$ (8)

Then, taking partial derivatives of L with respect to W, *H*_1_ and *H*_2_, we have

$$\frac{\partial L}{\partial W}=-(AH_{1}^{T}+\alpha SH_{2}^{T})+WH_{1}H_{1}^{T}+\alpha WH_{2}H_{2}^{T}+\psi$$ (9)

$$\frac{\partial L}{\partial H_{1}}=-W^{T}A+W^{T}WH_{1}+\beta H_{1}+\varphi$$ (10)

$$\frac{\partial L}{\partial H_{2}}=-{\alpha W}^{T}S+\alpha W^{T}WH_{2}+\beta H_{2}+\phi .$$ (11)

In terms of the Karush-Kuhn-Tucker (KKT) complementary slackness condition ψW = 0, φH_1_ = 0 and ϕH_2_ = 0, and Let $\frac{\partial L}{\partial W}=0$, $\frac{\partial L}{\partial H_{1}}=0$ and $\frac{\partial L}{\partial H_{2}}=0$, we can derive the following updating rules with respect to W, *H*_1_ and *H*_2_:

$$W⟵W.*(AH_{1}^{T}+\alpha SH_{2}^{T})./(WH_{1}H_{1}^{T}+\alpha WH_{2}H_{2}^{T})$$ (12)

$$H_{1}⟵H_{1}.*{(W}^{T}A)./{(W}^{T}WH_{1}+\beta H_{1})$$ (13)

$$H_{2}⟵H_{2}.*{(\alpha W}^{T}S)./{(\alpha W}^{T}WH_{2}+\beta H_{2})$$ (14)

where .* and ./ represent the elementwise multiplication and division, respectively. The score between nodes can be obtained by W and H_1_. Then, we can predict the edges.

To sum up, pseudo code of the proposed Link prediction algorithm based on NMF with coupling multivariate information is described as follows:

**Algorithm Name: SASNMF**

**Input**: A: the adjacency matrix of the given network, S: the

auxiliary information matrix, k: number of features, α and β: parameters.

**Output**: the approximate matrix of the network A

1: divide A into *A*^*train*^,*A*^*test*^

2: get the number of latent features k by Colibri

3: Initialize W, H_1_ and H_2_.

4: do while

5: update W, H_1_ and H_2_ by means of formulas (12),(13) and (14).

6: get W and H_1_ after until object function convergence

7: end while

8: output W × *H*_1_

### Computational complexity analysis

The computational complexity of SASNMF algorithm mainly comes from two parts. One is to extract auxiliary information, including external auxiliary information from node sociological attributes and internal auxiliary information extracted from topology structure. The second is iterative update matrices W, H_1_ and H_2_ at the same time.

Given an attributed network with n nodes, m attributes, then the matrix of attributes similarity, *S*_*n*×*n*_, is obtained by using cosine similarity algorithm based on node’s attribute vectors. So the time complexity is O(*n*^2^). Similarly, the time complexity of the internal auxiliary information extracted based on topology structure is also O(*n*^2^).

When updating W, H_1_ and H_2_, to reduce the time overhead, we utilizes the objective relative error as the stopping criterion and set to less than 10^−6^ in experiment. In addition, the decomposed dimension is a k-dimensional vector, their time complexities are O(*n*^2^k) time. So the total time cost of the algorithm is O(*n*^2^ + *n*^2^ + *n*^2^k). Since k can be treated as constants, complexity of the step is O(*n*^2^). To sum up, the computational cost of our approach is nearly to O(*n*^2^).

Of course, we can also improve our algorithm according to the relevant literature to achieve parallel computing[50], so as to obtain performance optimization. This is what we want to do in the future.

### Auxiliary information preprocessing

Here, we propose that the auxiliary information can be derived not only from external data but also from internal network structure information. SASNMF allows us to directly model such information into the framework to enhance the prediction performance. To distinguish sources of multivariate auxiliary information, we call those extracted from the network structure as ***internal*** auxiliary information and attributes of nodes as ***external*** auxiliary information.

It is an essential of our work that this external auxiliary information, node properties, is preprocessed. Considering the privacy of users, these information has been treated anonymously. When pretreated these attribute values, such as age, using directly actual measure values. Others, such as religious belief, are assigned a determined value in term of an appointed numerical range required. In addition, the numerical 0 or 1 is employed also to express two kinds of different status value. For these information, we use the vector Z_*m*_ to denote that the node has m attributes. All of the node’s attribute information in network G is represented as matrix Z_*n*×*m*_. The matrix element Z_*ij*_ represents the j^*th*^ attribute value of the *i*^*th*^ node. However, owing to the heterogeneity of node attribute, it is impossible that exert the better indicative effect of attributes on the prediction results through using a linear combination. Therefore, all of the attributes are normalized by the column of attribute matrix, that is, formula $Z_{ij}=\frac{Z_{ij}}{\sum_{k=1}^{n}Z_{kj}}$. Although it has been processed, the effectiveness of this attribute matrix in prediction is still very poor. Therefore, it is necessary to calculate the similarity between the attribute vectors Z_*m*_ of each node and to form the attribute similarity matrix before it can be applied to the prediction framework. To compute the similarity between attributes, the Euclidean distance, cosine similarity or Pearson method can be used to calculate. Here, the three common similarity measures were tested and analyzed respectively. Finally, we use the measure of similarity based on cosine, $S_{ij}=\frac{\sum_{l=1}^{m}Z_{il}{.Z}_{jl}}{\sqrt{\sum_{l=1}^{m}{Z_{il}}^{2}.\sum_{l=1}^{m}{Z_{jl}}^{2}}}$, to realize the evaluation of attribute similarity.

This internal auxiliary information is actually the latent feature of node, which the local structure information for the nodes themselves need be extracted from the input network by unsupervised structure similarity methods. In this work, for analysing the influence of node latent feature on the prediction performance, we employ seven similarity indices to compute the score, Sim, of the structure similarity between any two nodes as the internal auxiliary information. Furthermore, the prediction performance are analysed by comparing the node attribute with the structure information.

### Multivariate information combination mode

To test the effectiveness and analyse the influence to predict under different coupling modes of auxiliary information, we propose the following combination methods.

- A+S mode: the adjacent matrix A and external auxiliary information S are combined to input into the proposed framework. This method is directly marked as SASNMF.
- A+Sim mode: the adjacent matrix A and internal auxiliary information Sim are combined to input into the proposed framework. The Sim is regarded as matrix S in the proposed framework. Thus, this method is marked as *+SASNMF, where * represented any similarity methods.
- Sim+S mode: the adjacent matrix A is replaced as the internal auxiliary information Sim. This method is marked as A (= *)+SASNMF, where * represented any similarity methods.

For two types of network datasets: the second combination method, ii), is only used for the network without node attributes, while all of the methods are used for a network with real-world node attributes. Our experiments show that both types of auxiliary information can increase the performance of link prediction.

## Results

### Datasets description

We consider the following 13 real-world networks drawn from disparate fields. Among them, one contains external attributes, and we generate internal attributes for all of them.

The five networks with external attribute information: i) Lazega-lawyers [51]: The network is a social network between 71 partners and associates in some New England law firms. In addition, each entity in the network is described by features such as gender, office-location, age, and years employed. We did some preprocessing of the features (binarized the features such as the age and years employed) and then constructed a kernel matrix of pairwise similarities. In this article, we choose seven attributes to calculate. ii) Facebook [52]: The network is extracted from the Facebook online social network. A user can provide profile information (e.g., age, gender, education and information). By selecting some informative attributes in this profile information, we create a feature vector for each user. iii) WebKB [53]: The network consists of 4 subnetworks (Cornell, Texas, Washington and Wisconsin) gathered from 4 universities. The node represents a webpage that is annotated by 1703-dimensional binary valued word attributes. The first three of them are used for our experiments.

The eight networks without external attributes information: i) Karate [54]—social network of friendships between 34 members of a karate club at a US university in the 1970s; ii) Jazz [55]—jazz musician network, the link denotes the relationship between two persons if they played together in the same band; iii) USAir [56]—the air transportation network of US Airlines; iv) Political blogs (PolitB) [57]—the network of hyperlinks between weblogs on US politics; v) C. *elegans* [58]—the neural network of *C*. *elegans* worms; vi) Adjnoun [59]—The adjnoun network is the network of common adjectives and noun adjacencies for the novel “David Copperfield” by Charles Dickens; vii) Netsci [59]—Netsci is a collaboration network of researchers who publish papers on network science; and viii) Metabolic [58]—the metabolic network of the nematode worm *C*. *elegans*. These networks are often used as benchmark networks to test the predictive performance of new methods.

The basic topology features of these networks are summarized in Table 1. The symbol N and E are the total number of nodes and links, respectively. <K> is the average degree. <d> is the mean shortest distance. C is the clustering coefficient, and #attributes is the number of node attributes.

**Table 1:** The basic topology features of real networks.

Network	N	E	<K>	<d>	C	#attributes
**Lazega-lawyers**	71	378	10.8	2.104	0.391	7
**Facebook**	228	3419	29.991	1.868	0.616	56
**Cornell**	195	286	2.903	3.2	0.157	1703
**Texas**	187	298	3.027	3.036	0.196	1703
**Washington**	230	366	3.373	2.995	0.209	1703
**Krate**	34	78	4.588	2.408	0.571	/
**Jazz**	198	2742	27.70	2.235	0.618	/
**USAir**	332	2126	12.81	2.74	0.749	/
**PolitB**	1222	16714	27.36	2.74	0.36	/
**C. *elegans***	297	2148	14.47	2.46	0.308	/
**Netsci**	379	914	4.82	6.04	0.798	/
**Metabolic**	453	2025	8.940	2.664	0.647	/
**Adjnoun**	112	425	7.589	2.536	0.173	/

### Evaluation metrics

Like many existing prediction studies [1], in our work adopts also the most frequently-used metrics AUC (area under the ROC curve) to measure the performance of link prediction [60]. This metric is viewed as a robust measure in the presence of data imbalance [19].

The AUC can be interpreted as the probability that a randomly chosen missing link (a link in *E*^*test*^) is given a higher score than a randomly chosen nonexistent link (a link in U\E, where U denotes the universal set). In the implementation, among n independent comparisons, if there are *n*′ occurrences of the missing link having a higher score and *n*″ occurrences of the missing link and nonexistent link having the same score, we define the accuracy as:

$$AUC=\frac{n^{\prime }+0.5n^{\prime \prime }}{n}$$ (15)

If all the scores are generated from an independent and identical distribution, the accuracy should be approximately 0.5. Therefore, the degree to which the accuracy exceeds 0.5 indicates how much better the algorithm performs than pure chance.

In addition, we have adopted the Precision metric, which is also one of the most popular index of evaluation link prediction [61]. Given the ranking of the non-observed links in decreasing order according to their scores. The precision is defined as the ratio of relevant items selected to the number of items selected. That is to say, if we take the top-L links as the predicted ones, among which l links are right, then,

$$Precision=\frac{l}{L}$$ (16)

Clearly, a higher value of precision means a higher prediction accuracy.

Although the computing result is not unique through taking different *L* values for a single algorithm, in order to ensure the fairness for all comparison algorithms, the same value can be taken for *L*. This value does not affect the final comparison. Therefore, in our work, for the convenience of comparison, all the algorithms are unified to take the value of *L* = 100.

### Comparison methods

In this section, we mainly evaluate the performance of our algorithm. According to the way in multivariate information coupling mode, our methods are represented as SASNMF and *+SASNMF. More specifically, there are three types of coupling mode for auxiliary information using our framework, namely, i) Global network structure information coupling external auxiliary information from node attributes (A+S). ii) Global network structure information coupling internal auxiliary information from local structure latent feature (A+Sim). iii) Internal auxiliary information from local structure latent feature and external auxiliary information from node attributes are fused (Sim+S).

To analyse performance of algorithm proposed, we adopt two kinds of comparison methods. One is baseline algorithms, such as CN, AA, etc., which are often used for existing methods as benchmark to evaluate these approaches. We used seven here. In this work, they are also used to extract local structural latent features of nodes to act as internal auxiliary information.

The second is several state-of-the-art methods. These are divided into two categories: both structural information and node attribute information are adopted and only structural information is utilized.

### Baseline methods

We list four types of link prediction methods as the baseline methods, including five local algorithms based on the number of common neighbours between pairs of nodes (CN,AA,RA,Salton and Jaccard), a global random walk method(ACT) and a local path method(Katz) and NMF method based on matrix factorization with the Frobenius norm. The mathematical expressions of these methods are shown in Table 2. Their detailed definitions can be found in ref. 1–3 and 43.

**Table 2:** Mathematical expressions of baseline methods.

Methods	Formula	Notes
Common neighbour(CN)	*S*_*xy*_ = |Γ(*x*) ∩ Γ(*y*)|	Where Γ(*x*) denotes the set of neighbours of node x, |*| is the cardinality of the set *, and *k*(*x*) is the degree of node *x*.
Salton	$S_{xy}=\frac{\left|\Gamma (x)\cap \Gamma (y)\right|}{\sqrt{k(x)\times k(y)}}$	
Jaccard	$S_{xy}=\frac{\left|\Gamma (x)\cap \Gamma (y)\right|}{\left|\Gamma (x)\cup \Gamma (y)\right|}$	
Resource Allocation Index(RA)	$S_{xy}=\sum_{Z\in \Gamma (x)\cap \Gamma (y)}\frac{1}{k(Z)}$	
Adamic-Adar index(AA)	$S_{xy}=\sum_{Z\in \Gamma (x)\cap \Gamma (y)}\frac{1}{logk(Z)}$	
Average Commute Time (ACT)	$S_{xy}=\frac{1}{l_{xx}^{+}+l_{yy}^{+}-2l_{xy}^{+}}$	Where $l_{xy}^{+}$ represents the elements of matrix *L*^+^, the pseudo-inverse of the Laplacian matrix.
Katz	*S*_*xy*_ = ((*I* − *θ* ∙ *A*)^−1^ − *I*)_*xy*_	Where *θ* is a parameter, takes the default value 0.1, and *I* is the diagonal matrix.
NMF	Non-negative matrix factorization	MF-based method

### State-of-the-art methods

In addition, apart from the baseline methods, we also further compare the performance of the proposed SASNMF method with the other three state-of-art competitive algorithms.

The structure perturbation method (SPM) based on nonnegative matrix factorization [24], which is based on the perturbation of the adjacency matrix, assumes that the regularity of a network is reflected in the consistency of structural features before and after a random removal of a small set of links. In particular it outperforms state-of-the-art link prediction methods both in accuracy and robustness[22,23]. In the SPM method, we use the method of NMF-D1 with random deletion perturbation. And the perturbation ratio is 0.04, the default value of perturbation times is 20.

Matrix completion (MC) [25] is a global information-based prediction algorithm based upon the low-rank and sparse property of the adjacency matrix. It employ the robust principal component analysis method through minimizing the nuclear norm of the matrix which fits the training data to reconstruct a network that is close to the original network and accordingly identify the missing links. In the MC method, in addition to the partial values of the parameter λ provided in the literature, we also perform an optimal analysis of the parameter and finally select the best one. The parameter values of this method are referred to in the **S1 File.**

In addition, Chen BL et al. [41] proposed a link prediction method based on NMF(NMF-LP), which adopted node attributes. Therefore, we compare this method with our framework.

### Experiments results

**Fig 1:**
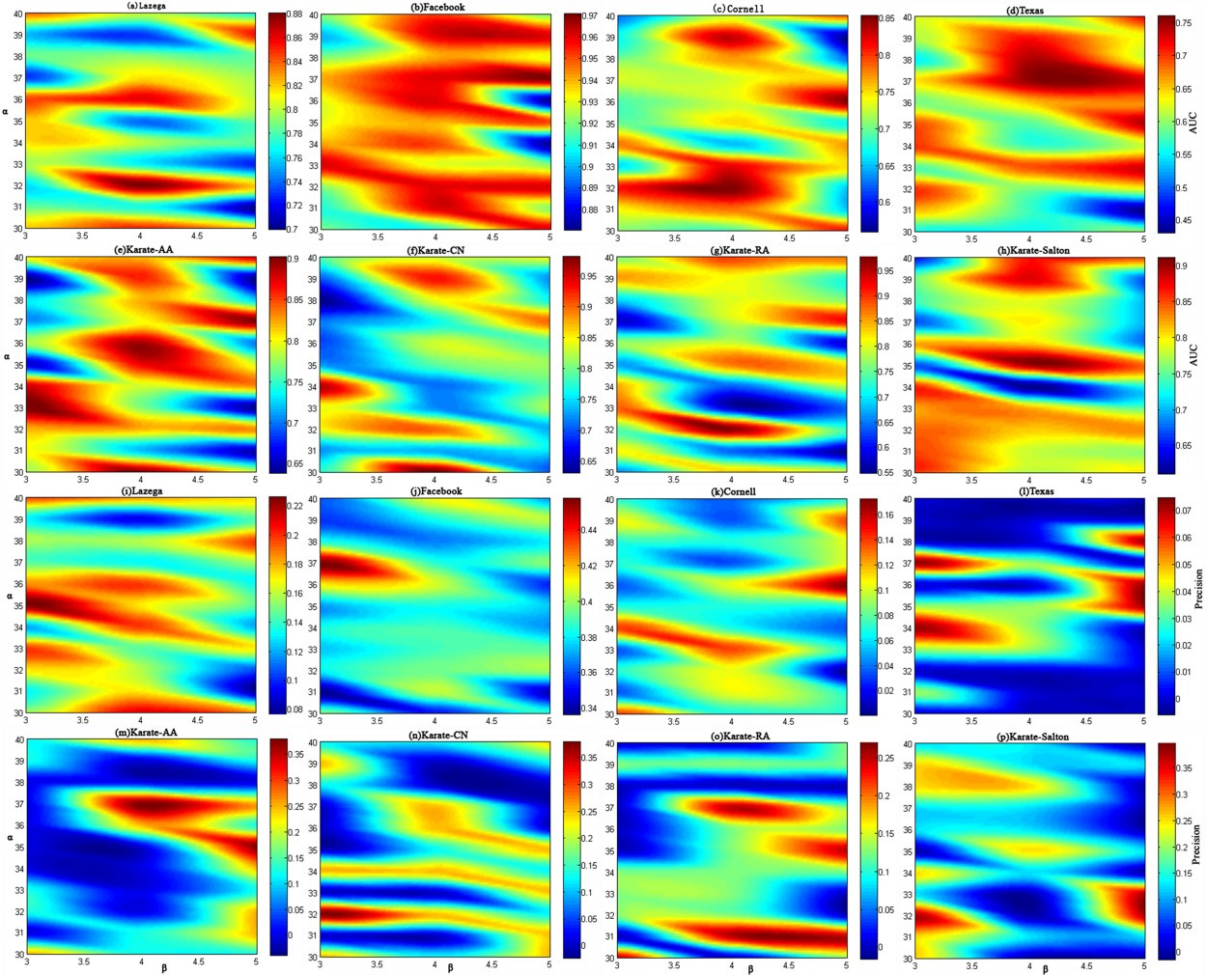
Model parameter sensitivity analysis.

Parameters setting: In order to achieve good prediction results, before the whole experiment, we analyzed the sensitivity of the model parameters α and β. We set the proportion of training set as 0.9, and the range of the two parameters are set from 1 to 100, respectively. And then take the widely used evaluation index AUC and Precision for link predication as evidence. The values of AUC and precision are calculated on 13 networks, and compared with each other. Finally, the optimal range of parameters is gradually obtained. Furthermore, we select five networks including Lazega, Facebook, Cornell, Texas, four networks with node attributes and Kate, one non-attributes from the all networks, and analyze the experimental sensitivity of α and β in the performance of link predication in a smaller range. As represented in Fig1, it is obvious that the performances on Lazega, Facebook, Cornell, Texas and Kate are gradual stable. Although the different settings of α and β have significant influence on the predict results, we also know that our framework has equally better performance than other baseline methods. Without losing generality, we set α = 4, β = 32 in subsequent experiments.

Using optimized parameter results, in this section, we show the AUC and precision results of our proposed methods based on NMF with coupling multivariate information and other comparison methods on the 13 real network data in Tables 3–6.

**Table 3:** The average predicting precision obtained by 100 independent runs on 5 networks with external attributes. The training set contains 90% of the total connections.

Precision	Lazega	Facebook	Cornell	Texas	Washington	Mean	Mode
SASNMF	0.1661^(2)^	0.3923^(1)^	0.0655^(12)^	0.0154^(15)^	0.0451^(7)^	7.4	A+S
AA+SASNMF	0.1579^(3)^	0.2952^(10)^	0.0917^(6)^	0.0182^(11)^	0.0092^(14)^	8.8	A+Sim
CN+SASNMF	0.1479^(6)^	0.2913^(13)^	0.0934^(5)^	0.0168^(14)^	0.0114^(12)^	10	
RA+SASNMF	0.1516^(4)^	0.2931^(12)^	0.0866^(9)^	0.0193^(10)^	0.0108^(13)^	9.6	
Salton+SASNMF	0.1484^(5)^	0.2963^(9)^	0.0876^(7)^	0.0171^(13)^	0.0146^(11)^	9	
A (= AA)+SASNMF	0.1316^(12)^	0.2836^(16)^	0.1069^(3)^	0.1071^(1)^	0.1135^(1)^	6.6	Sim+S
A (= CN)+ SASNMF	0.1474^(7)^	0.2842^(15)^	0.0828^(10)^	0.0536^(5)^	0.0919^(3)^	8	
A (= RA)+ SASNMF	0.1316^(12)^	0.2944^(11)^	0.1103^(2)^	0.0857^(2)^	0.1000^(2)^	5.8	
A (= Salton)+ SASNMF	0.0842^(18)^	0.1646^(19)^	0.0000^(18)^	0.0000^(18)^	0.0000^(15)^	17.6	
AA	0.1321^(11)^	0.3247^(4)^	0.0869^(8)^	0.0739^(3)^	0.0873^(5)^	6.2	Baseline methods
CN	0.1371^(10)^	0.3136^(7)^	0.0741^(11)^	0.0432^(6)^	0.0892^(4)^	7.6	
RA	0.1271^(14)^	0.3808^(2)^	0.0866^(9)^	0.0700^(4)^	0.0792^(6)^	7	
Salton	0.0953^(16)^	0.3002^(8)^	0.0000^(18)^	0.0004^(17)^	0.0000^(15)^	14.8	
Jaccard	0.0921^(17)^	0.3162^(6)^	0.0010^(17)^	0.0004^(17)^	0.0000^(15)^	14.4	
Katz	0.1303^(13)^	0.0163^(20)^	0.0359^(15)^	0.0104^(16)^	0.0222^(8)^	14.4	
ACT	0.0311^(19)^	0.2575^(17)^	0.0255^(16)^	0.0179^(12)^	0.0000^(15)^	15.8	
NMF	0.1471^(8)^	0.2907^(14)^	0.0969^(4)^	0.0154^(15)^	0.0108^(13)^	10.8	
SPM	0.1742^(1)^	0.3546^(3)^	0.1276^(1)^	0.0314^(8)^	0.0200^(10)^	4.6	State-of-the-art methods
MC	0.1084^(15)^	0.3184^(5)^	0.0455^(14)^	0.0400^(7)^	0.0200^(9)^	10	
NMF-LP	0.1461^(9)^	0.1715^(18)^	0.0621^(13)^	0.0243^(9)^	0.0146^(11)^	12	

**Table 4:** The average predicting AUC obtained by 100 independent runs on 5 real networks with external attributes. The training set contains 90% of the total connections.

AUC	Lazega	Facebook	Cornell	Texas	Washington	Mean	Mode
SASNMF	0.8003^(4)^	0.9354^(3)^	0.7000^(9)^	0.6398^(15)^	0.6886^(10)^	8.2	A+S
AA+SASNMF	0.7717^(9)^	0.9075^(11)^	0.7830^(4)^	0.6734^(8)^	0.7368^(5)^	7.4	A+Sim
CN+SASNMF	0.7668^(13)^	0.9088^(9)^	0.7875^(3)^	0.6686^(10)^	0.7358^(6)^	8.2	
RA+SASNMF	0.7704^(11)^	0.9137^(8)^	0.7876^(2)^	0.6730^(9)^	0.7410^(3)^	6.6	
Salton+SASNMF	0.7707^(10)^	0.9138^(7)^	0.7817^(5)^	0.6746^(7)^	0.7378^(4)^	6.6	
A (= AA)+SASNMF	0.7960^(5)^	0.8810^(14)^	0.7000^(9)^	0.7060^(3)^	0.7330^(7)^	7.6	Sim+S
A (= CN)+ SASNMF	0.8030^(2)^	0.8580^(15)^	0.6600^(15)^	0.6490^(12)^	0.6650^(13)^	11.4	
A (= RA)+ SASNMF	0.8120^(1)^	0.8950^(13)^	0.7270^(8)^	0.7170^(2)^	0.7700^(1)^	5	
A (= Salton)+ SASNMF	0.7350^(17)^	0.8210^(18)^	0.6500^(16)^	0.5590^(18)^	0.6310^(16)^	17	
AA	0.7864^(7)^	0.9355^(2)^	0.6973^(11)^	0.6807^(5)^	0.6919^(9)^	6.8	Baseline methods
CN	0.7768^(8)^	0.9243^(6)^	0.6673^(14)^	0.6489^(13)^	0.6609^(14)^	11	
RA	0.7896^(6)^	0.9514^(1)^	0.6956^(12)^	0.6748^(6)^	0.6925^(8)^	6.6	
Salton	0.7587^(14)^	0.9260^(5)^	0.6179^(18)^	0.5765^(17)^	0.6081^(17)^	14.2	
Jaccard	0.7559^(15)^	0.9067^(12)^	0.6188^(17)^	0.5794^(16)^	0.6063^(18)^	15.6	
Katz	0.5876^(20)^	0.3394^(20)^	0.6792^(13)^	0.3392^(20)^	0.3898^(20)^	18.6	
ACT	0.6485^(18)^	0.8468^(16)^	0.7341^(7)^	0.7002^(4)^	0.6513^(15)^	12	
NMF	0.7673^(12)^	0.9086^(10)^	0.7639^(6)^	0.6650^(11)^	0.6868^(11)^	10	
SPM	0.8014^(3)^	0.9294^(4)^	0.8063^(1)^	0.7274^(1)^	0.7615^(2)^	2.2	State-of-the-art methods
MC	0.6072^(19)^	0.8326^(17)^	0.5068^(19)^	0.4354^(19)^	0.4770^(19)^	18.6	
NMF-LP	0.7551^(16)^	0.7795^(19)^	0.6975^(10)^	0.6401^(14)^	0.6705^(12)^	14.2	

**Table 5:** The average predicting precision obtained by 100 independent runs on 8 real networks with only internal attributes. The training set contains 90% of the total connections.

Precision	Karate	Jazz	USAir	PolitB	C.*elegans*	NetSci	Metabolic	Adjnoun	Mean	Mode
AA+SASNMF	0.1575^(4)^	0.5519^(7)^	0.3387^(6)^	0.1829^(2)^	0.1432^(5)^	0.3595^(7)^	0.2630^(3)^	0.0684^(4)^	4.75	A+Sim
CN+SASNMF	0.1600^(3)^	0.5563^(5)^	0.2087^(10)^	0.1142^(10)^	0.1417^(6)^	0.3247^(10)^	0.1758^(9)^	0.0279^(8)^	7.625	
RA+SASNMF	0.1525^(5)^	0.5570^(4)^	0.2051^(11)^	0.1185^(9)^	0.1459^(4)^	0.3555^(8)^	0.1797^(7)^	0.0272^(9)^	7.125	
Salton+SASNMF	0.1725^(2)^	0.5588^(3)^	0.3096^(8)^	0.1455^(7)^	0.1466^(3)^	0.3306^(9)^	0.2308^(4)^	0.0329^(7)^	5.375	
AA	0.1267^(9)^	0.5234^(10)^	0.3991^(3)^	0.1735^(4)^	0.1057^(8)^	0.7192^(2)^	0.1969^(6)^	0.0767^(2)^	5.5	Baseline methods
CN	0.1150^(11)^	0.5031^(12)^	0.3786^(4)^	0.1748^(3)^	0.0913^(10)^	0.5062^(5)^	0.1410^(10)^	0.0726^(3)^	7.25	
RA	0.1371^(7)^	0.5413^(8)^	0.4683^(1)^	0.1504^(6)^	0.1029^(9)^	0.7312^(1)^	0.2726^(2)^	0.0649^(5)^	4.875	
Salton	0.0008^(14)^	0.5314^(9)^	0.0521^(14)^	0.0102^(14)^	0.0182^(14)^	0.5496^(3)^	0.0510^(12)^	0.0014^(12)^	11.5	
Jaccard	0.0013^(13)^	0.5176^(11)^	0.0677^(12)^	0.0167^(13)^	0.0207^(13)^	0.5489^(4)^	0.0495^(13)^	0.0016^(11)^	11.25	
Katz	0.1358^(8)^	0.0202^(14)^	0.0527^(13)^	0.0265^(12)^	0.0222^(12)^	0.0995^(13)^	0.0192^(14)^	0.0009^(13)^	12.375	
ACT	0.1088^(12)^	0.1679^(13)^	0.3304^(7)^	0.0740^(11)^	0.0533^(11)^	0.0000^(14)^	0.0934^(11)^	0.0967^(1)^	10	
NMF	0.1488^(6)^	0.5548^(6)^	0.2111^(9)^	0.1213^(8)^	0.1493^(2)^	0.3189^(11)^	0.1796^(8)^	0.0235^(10)^	7.5	
SPM	0.2250^(1)^	0.6092^(2)^	0.3677^(5)^	0.1711^(5)^	0.1702^(1)^	0.4801^(6)^	0.2888^(1)^	0.0386^(6)^	3.375	State-of-the-art methods
MC	0.1163^(10)^	0.6143^(1)^	0.4205^(2)^	0.1872^(1)^	0.1256^(7)^	0.3068^(12)^	0.2179^(5)^	0.0279^(8)^	5.75	

**Table 6:** The average predicting AUC obtained by 100 independent runs on 8 real networks with only internal attributes. The training set contains 90% of the total connections.

AUC	Karate	Jazz	USAir	PolitB	C.*elegans*	NetSci	Metabolic	Adjnoun	Mean	Mode
AA+SASNMF	0.7721^(2)^	0.9598^(6)^	0.9502^(5)^	0.9420^(1)^	0.8723^(2)^	0.9350^(8)^	0.8652^(4)^	0.7143^(2)^	3.75	A+Sim
CN+SASNMF	0.7361^(6)^	0.9534^(11)^	0.8987^(10)^	0.7980^(12)^	0.8332^(7)^	0.9401^(6)^	0.7979^(9)^	0.6213^(10)^	8.875	
RA+SASNMF	0.7217^(9)^	0.9570^(8)^	0.8941^(11)^	0.8253^(11)^	0.8256^(8)^	0.9338^(9)^	0.7923^(10)^	0.6171^(12)^	9.75	
Salton+SASNMF	0.7688^(3)^	0.9538^(10)^	0.9472^(6)^	0.8940^(7)^	0.8588^(5)^	0.9359^(7)^	0.8329^(6)^	0.6800^(7)^	6.375	
AA	0.7282^(8)^	0.9664^(3)^	0.9684^(2)^	0.9270^(2)^	0.8654^(4)^	0.9916^(2)^	0.9561^(2)^	0.6866^(5)^	3.5	Baseline methods
CN	0.6984^(10)^	0.9591^(7)^	0.9550^(3)^	0.9213^(4)^	0.8423^(6)^	0.9904^(5)^	0.9236^(3)^	0.6898^(4)^	5.25	
RA	0.7338^(7)^	0.9721^(1)^	0.9734^(1)^	0.9265^(3)^	0.8695^(3)^	0.9908^(4)^	0.9607^(1)^	0.6819^(6)^	3.25	
Salton	0.6321^(12)^	0.9667^(2)^	0.9254^(7)^	0.8782^(8)^	0.7874^(11)^	0.9931^(1)^	0.8119^(7)^	0.6202^(11)^	7.375	
Jaccard	0.6068^(13)^	0.9619^(5)^	0.9178^(8)^	0.8752^(9)^	0.7924^(10)^	0.9915^(3)^	0.7808^(11)^	0.6257^(9)^	8.5	
Katz	0.7475^(4)^	0.4076^(14)^	0.3843^(14)^	0.4766^(14)^	0.4722^(14)^	0.9206^(10)^	0.4535^(14)^	0.2607^(14)^	12.25	
ACT	0.6603^(11)^	0.7973^(13)^	0.8990^(9)^	0.9006^(6)^	0.7548^(12)^	0.5758^(14)^	0.7654^(12)^	0.7462^(1)^	9.75	
NMF	0.7387^(5)^	0.9556^(9)^	0.8761^(12)^	0.8395^(10)^	0.8250^(9)^	0.9039^(12)^	0.8008^(8)^	0.6352^(8)^	9.125	
SPM	0.7978^(1)^	0.9624^(4)^	0.9504^(4)^	0.9132^(5)^	0.8766^(1)^	0.9110^(11)^	0.8482^(5)^	0.7082^(3)^	4.25	State-of-the-art methods
MC	0.5704^(14)^	0.8709^(12)^	0.8142^(13)^	0.6767^(13)^	0.5874^(13)^	0.6721^(13)^	0.6026^(13)^	0.4670^(13)^	13	

Tables 3 and 4 show the results calculated on five networks with external auxiliary information (namely, node attributes), while Tables 5 and 6 show the eight networks with only internal information. To facilitate comparison, we add Mode column to the table, and classify it according to different combination mode and different comparison method to show the difference. In the four tables, the presented links for every dataset are partitioned into a training set (90%) and a probe set (10%). From these tables, we can see that the prediction results by means of various combination formulas under the SASNMF framework are significantly better than the other comparison methods. In addition, these methods using external auxiliary information are generally superior to the baseline methods that use only structure information.

These experimental results are classified according to whether the network has external auxiliary information, namely, node attributes, and both AUC and precision evaluation criteria were used for performance analysis. In the four tables, the upper right of the numbers represents the respective Precision-ranking (AUC-ranking) position of each method in each network. The smaller the number is, the better the prediction performance of the algorithm (see **S1 File**). To reflect the overall performance of all algorithms on different networks, the column labelled as Mean in the table is the mean ranking value of each method across all the networks. It is an indicator of average performance. To facilitate analysis, the column labelled as Mode represents different information combinations. Through the results shown in these four tables, we can see that although the methods proposed: A+S, A + Sim, Sim + S were not always the best, it can be found from the average of performance ranking levels on each network that the prediction performance of these three forms based on the SASNMF framework are in the leading position as a whole. This finding indicates that this auxiliary information, including the internal structure latent features and the external node attributes, is salutary to enhance the accuracy of link prediction.

**Fig 2:**
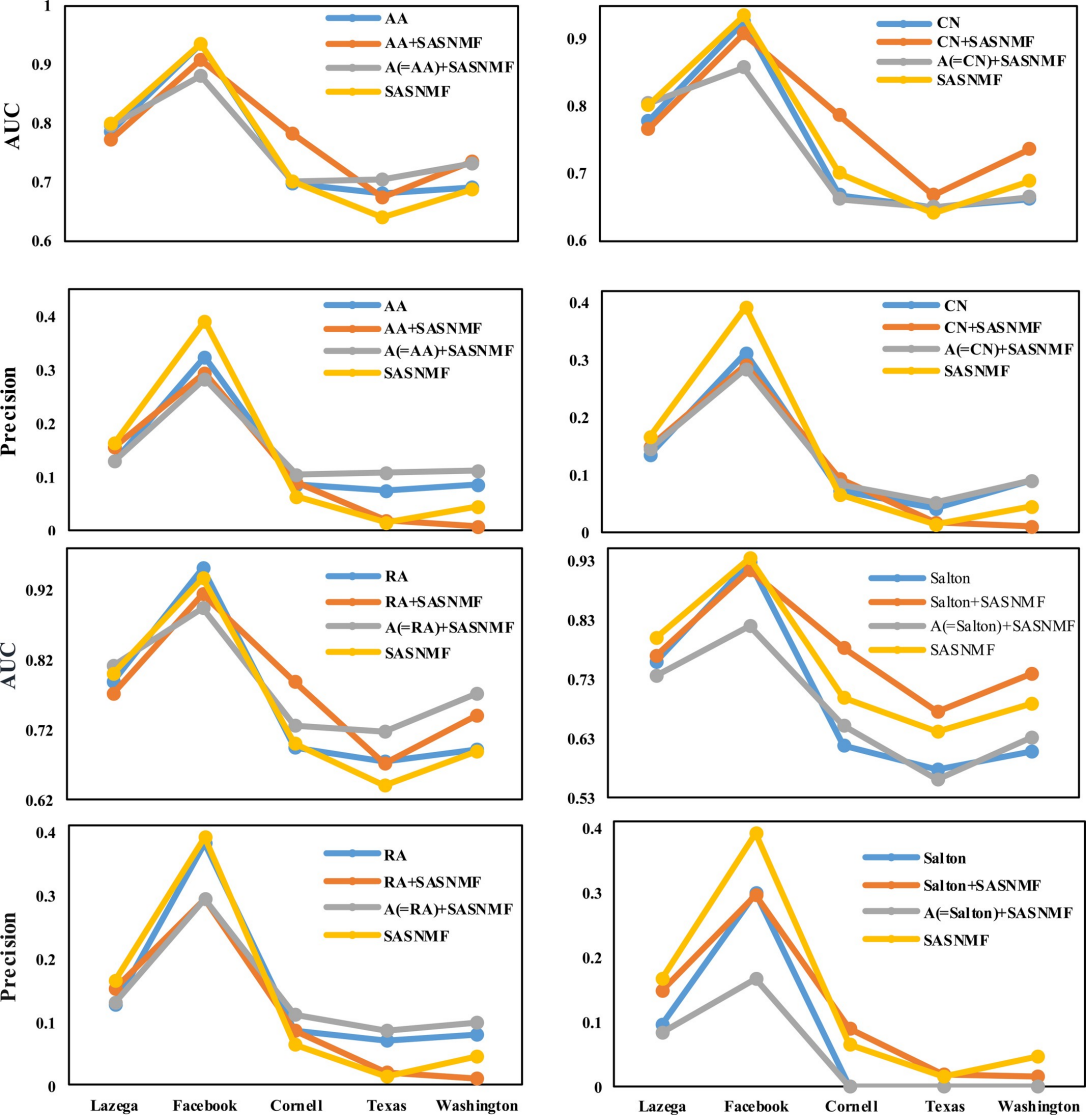
The AUC and precision score on 5 real networks with external attribute information.

To further test the overall prediction effect of the three combination methods proposed, we give only the results of precision and AUC based on four baseline methods, AA, CN, RA and Salton on real networks in Fig 2. Here, we use a baseline method and its two combinations, namely, A+Sim and Sim+S, to compare with SASNMF.

**Fig 3:**
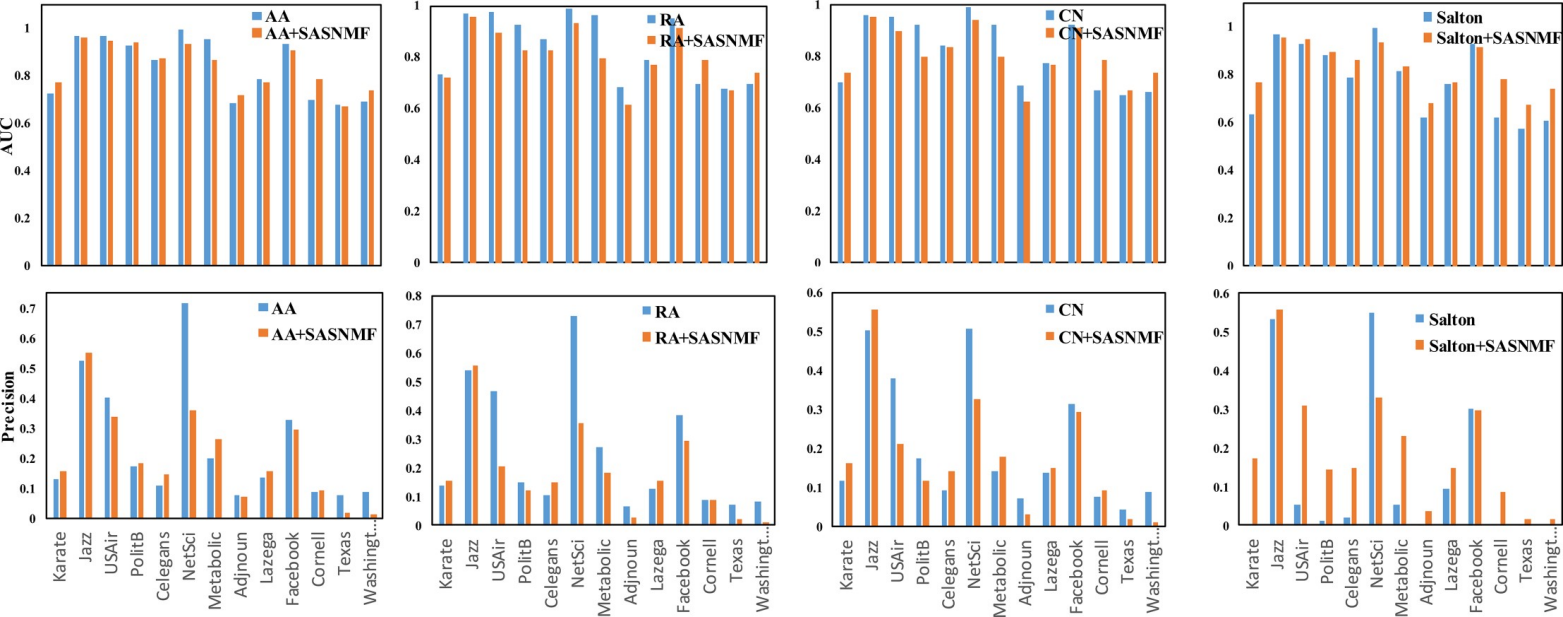
The AUC and precision results compared with baseline methods on 13 real networks.

**Fig 4:**
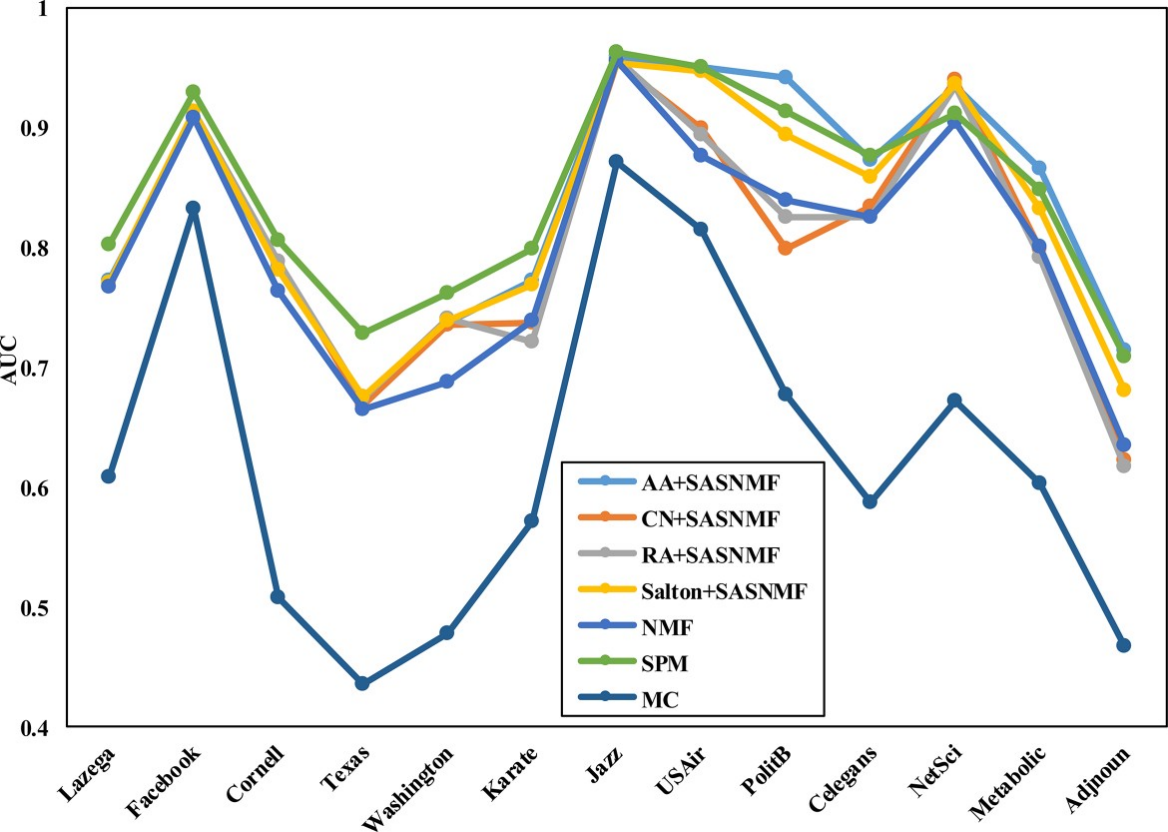
The AUC results compared with the state-of-the-art methods on 13 real networks.

Similarly, to compare the overall performance of the combined mode A+Sim with the baseline method and the state-of-the-art methods on 13 real networks, we consider four baseline methods (AA, CN, RA and Salton) and their combined modes. The AUC and precision results are shown in Figs 3 and 4.

From Fig 4, we can see that the proposed combination method based on our framework is also better overall than the MC and NMF methods besides the SPM. Of course, the SPM method is not as good as our method on some of the datasets in the experiment.

**Fig 5:**
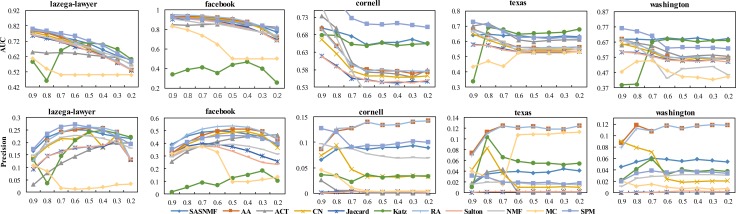
The precision and AUC results in different proportion training sets.

In addition, to test the performance of our methods, the relative precision and AUC results of our proposed methods and other baseline methods under different fractions of training sets in the different network are shown in Fig 5.

For the NMF-LP method, because it is a link prediction method based on node attribute information, we only make a comparative analysis with it on these networks with node attributes. In the whole comparative experiment, we find that the time complexity of NMF-LP method is much higher than our algorithm, and from the final experimental results, the performance of our algorithm is more competitive than it.

## Discussion

In summary, real networks are sparse and contain noise. To overcome prediction difficulties by means of internal and external auxiliary information, we proposed a unified prediction framework based on non-negative matrix factorization with coupling multivariate information, which can model the internal latent feature information and external node attribute information of the network. Based on this framework, we also proposed three combination methods that are represented as A+S, A+Sim, and Sim+S. According to the proposed combination patterns, we design a large number of experiments for networks with node attributes and networks without node attributes under our framework. We compared the proposed methods with 8 benchmark methods and 3 state-of-the-art methods on 13 real network datasets.

**Fig 6:**
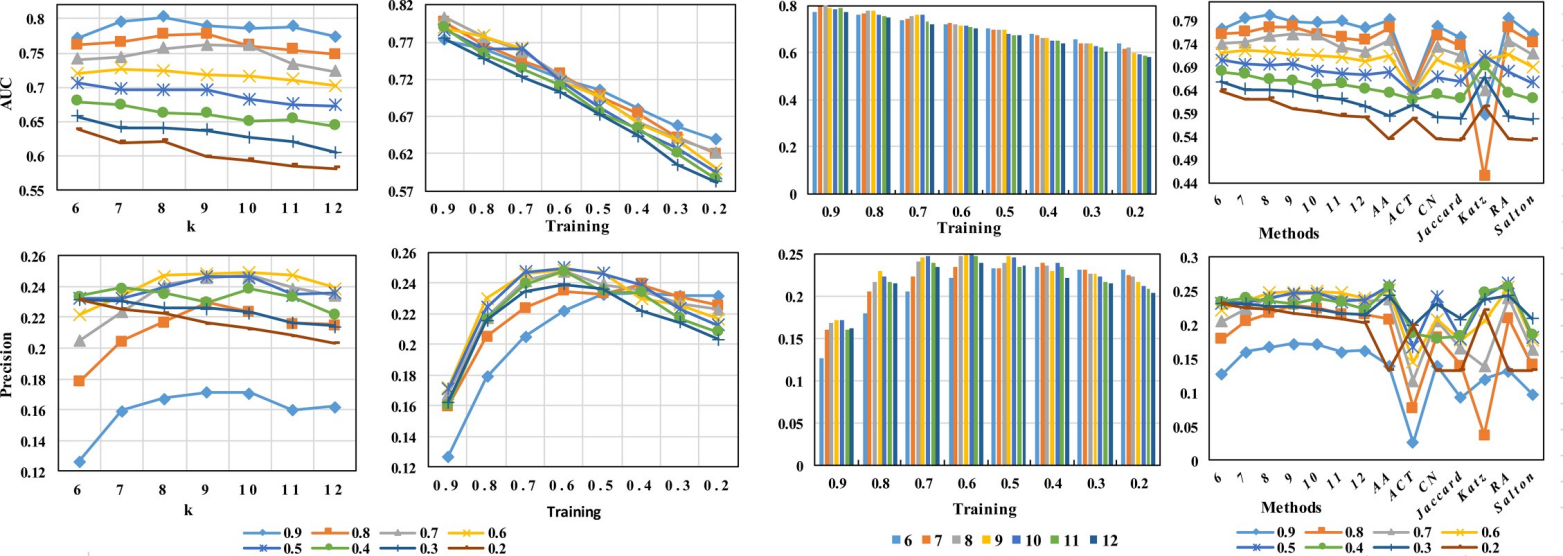
The accuracy of different k values is calculated and compared by two metrics.

In addition, the selection of the rank after the matrix decomposition was also important because of its effect on the prediction result and the number of latent features k in the SASNMF framework is different for each dataset. Here, to illustrate the problem, the results of different k for the Lazega-lawyer dataset are shown as follows in Fig 6.

In the figure, the training sets are from 90% to 20% and only a network dataset—Lazega-lawyer.

As seen in Figs 2 and 3, the methods in which the mode is A+S, A+Sim and Sim+S are better than the corresponding benchmark methods. Especially, through our framework, the prediction effect of using node attributes as auxiliary information is competitive compared to those baseline methods.

To better test the extensibility and robustness, Fig 5 shows the results of precision and AUC under different proportions of training sets E^*train*^ and test sets E^*test*^. Fig 5 shows a prediction trend for five attribute networks, where the partition ratio, E^*train*^ and E^*test*^, is from 0.9 to 0.2. We find that the performance of all methods declines obviously as the E^*train*^ ratio decreases in Fig 5. However, there is a gentle trend decline under the SASNMF method. Moreover, from the whole process of dataset partitioning to analyse the results synthetically, its prediction effect is obviously superior to other baseline methods. This finding indicates that these methods that rely only on structural information can make the prediction worse as the number of connected sets in the training set decreases. Our framework can alleviate the problem of data sparsity by coupling multivariate auxiliary information. Especially, on the Lazega-lawyer and Facebook datasets, the impact of using SASNMF on the results is obviously better than that of other comparison methods. Although the precision test of the Cornell, Texas and Washington datasets is inferior to that of AA and RA, our model is far better than that of these two methods under the corresponding AUC evaluation. It can be said that the overall effect of our method is good under the AUC index.

Therefore, why does our method not work well on these three datasets? Through in-depth analysis, we think that the main reason for this phenomenon lies in the attribute information. In fact, the attribute values used in these three datasets are simply quantized whether the words in the article appear or not, compared with the first two data sets. However, the attribute values of the first two datasets are true social attributes. Therefore, the attribute of these three networks cannot be said to better reflect the true similarity between nodes.

In addition, the number of latent features k in the SASNMF framework is different for each dataset. Moreover, the determination of the latent features k is a very important and difficult problem in matrix factorization. Fig 6 shows only the results under different k for the Lazega-lawyer dataset. In this paper, because it is not our primary focus, we take an easy and effective method for automatic determination of k, by Colibri [62], which seeks a nonorthogonal basis by sampling the columns of the input matrix. However, to observe the influence of different k in the process of matrix factorization for the prediction effect, we take some of k’s value by means of the limitative form of k(m + n) ≪ mn provisionally. Due to the adjacent matrix A being symmetrical here, the k is far less than n/2. Fig 6 shows that the influence of the selection of k on the prediction results is obvious.

## Conclusion

In recent years, link prediction based on network topology has been one of the research hotspots in the field of data mining. However, in many instances, algorithms that use only network structure do not provide the precision needed for link prediction. At present, with the development of mobile Internet, the more descriptive information owned by the entities in the network is becoming an asset to be used. Inspired by this, based on the advantages of NMF such as interpretability, nonnegativity and information fusion, a unified framework of link prediction is proposed in this paper. By this framework, the adjacency matrix A, which represents the macroscopic information of a network topology, and the auxiliary information matrix S, which represents the microscopic information of the network, are mapped to the same low-rank latent feature space to realize the multivariate information coupling. Then, the link prediction task can be realized by merging into a prediction matrix that can infer the missing relationship of the network. At the same time, to further analyse the usability of the network auxiliary information, we not only use the external attributes of the nodes but also explore the latent features of the nodes that are extracted as internal auxiliary information by some traditional structural similarity indices from local and global perspectives. On the basis of multivariate information, we further propose three different combinations. We used three class combination forms as the simulation cases of the proposed framework and experiments to show the feasibility, effectiveness, and competitiveness of the framework. Moreover, a large number of experiments on five networks with node sociological attributes and eight networks without node attributes show that the prediction performance under this unified framework is competitive compared with seven baseline methods and three state-of-art methods on the whole according to the different combination patterns proposed by us. This finding demonstrates that the proposed framework has advantages in combining the structure and attribute information for link prediction. Furthermore, the framework is easy to extend to directed and weighted networks by letting the matrix V be directed and weighted because it is based on NMF.

In the future, there are some limitations and improved studies for our proposed framework. One of which is how to set parameters α and β to be adaptive on different networks. Furthermore, we will extend our methods to more generalized situations such as extending the model to edge attributes and combination attributes of edges and nodes and dynamic network link prediction. Designing efficient methods to solve these issues will be interesting.

## Supporting information

S1 File This is the data source for Figs 4 and 5. (XLSX)

## References

[1] Lü LY, Zhou T. Link prediction in complex networks: A survey. Physica A Statistical Mechanics & Its Applications, 2011, 390(6):1150–1170. 10.1016/j.physa.2010.11.027

[2] Wang P, Xu BW, Wu YR, Zhou XY. Link prediction in social networks: the state-of-the-art. Science China Information Sciences, 2015, 58(1):1–38. 10.1007/s11432-014-5237-y

[3] Martínez V, Berzal F, Cubero J C. A Survey of Link Prediction in Complex Networks. Acm Computing Surveys, 2017, 49(4):69 10.1145/3012704

[4] kumar R, Novak J, Tomkins A. Structure and evolution of online social networks. KDD’06, August 20–23, 2006, Philadelphia, Pennsylvania, USA.

[5] Liu Z, Zhang Q M, Lü LY, Zhou T. Link prediction in complex networks: a local naïve Bayes model. Europhysics Letters, 2011, 96(4): 48007 10.1209/0295-5075/96/48007

[6] Guan Q, An HZ, Gao XY, Huang SP, Li HJ. Estimating potential trade links in the international crude oil trade: A link prediction approach. Energy, 2016, 102:406–415. 10.1016/j.energy.2016.02.099

[7] Cheng ZY, Caverlee J, Lee K, Sui DZ. Exploring Millions of Footprints in Location Sharing Services. Proceedings of the Fifth International AAAI Conference on Weblogs and Social Media, 2011: 81–88.

[8] Feng SS, Li XT, Zeng YF, C G, Chee YM, Y Q. Personalized ranking metric embedding for next new POI recommendation. International Conference on Artificial Intelligence. AAAI Press, 2015:2069–2075.

[9] Bohannon John. Counterterrorism's new tool: 'metanetwork' analysis. Science, 2009, 325(5939):409–411. 10.1126/science.325_409 19628852

[10] Benigni MC, Joseph K, Carley KM. Online extremism and the communities that sustain it: Detecting the ISIS supporting community on Twitter. Plos One, 2017, 12(12):e0181405 10.1371/journal.pone.0181405 29194446 PMC5711025

[11] Tayebi M A, Glässer U. Social Network Analysis in Predictive Policing. Springer press, 2016 10.1007/978-3-319-41492-8_2

[12] Budur E, Lee S, Kong VS. Structural Analysis of Criminal Network and Predicting Hidden Links using Machine Learning. Computer Science, 2015:641–650.

[13] Berlusconi G, Calderoni F, Parolini N, Verani M, Piccardi C. Link Prediction in Criminal Networks: A Tool for Criminal Intelligence Analysis. Plos One, 2016, 11(4):e0154244 10.1371/journal.pone.0154244 27104948 PMC4841537

[14] Liben-Nowell D, Kleinberg J. The Link Prediction Problem for Social Networks. Journal of the American Society for Information Science and Technology, 2007, 58(7):1019–1031. 10.1002/asi.v58:7

[15] Jordan T, Alves OCP, Wilde PD, Lima-Neto FBD. Link-prediction to tackle the boundary specification problem in social network surveys. Plos One, 2017, 12(4):e0176094 10.1371/journal.pone.0176094 28426826 PMC5398605

[16] Tsugawa S, Kito K. Retweets as a Predictor of Relationships among Users on Social Media. Plos One, 2017, 12(1):e0170279 10.1371/journal.pone.0170279 28107489 PMC5249064

[17] Hasan M A, Chaoji V, Salem S, Zaki M. Link prediction using supervised learning. Proc of Sdm Workshop on Link Analysis Counterterrorism & Security, 2006, 30(9):798–805.

[18] Menon A K, Elkan C. A Log-Linear Model with Latent Features for Dyadic Prediction. IEEE, International Conference on Data Mining. IEEE, 2011:364–373. 10.1109/ICDM.2010.148

[19] Menon AK, Elkan C. Link prediction via matrix factorization. European Conference on Machine Learning and Knowledge Discovery in Databases. Springer-Verlag, 2011:437–452. 10.1007/978-3-642-23783-6_28

[20] Clauset A, Moore C, Newman M E. Hierarchical structure and the prediction of missing links in networks. Nature, 2008, 453(7191):98–101. 10.1038/nature06830 18451861

[21] Pan L, Zhou T, Lü LY, Hu CK. Predicting missing links and identifying spurious links via likelihood analysis. Scientific Reports, 2016, 6:22955 10.1038/srep22955 26961965 PMC4785364

[22] Lü LY, Pan LM, Zhou T, Zhang YC, Stanley H E. Toward link predictability of complex networks. Proceedings of the National Academy of Sciences of the United States of America, 2015, 112(8):2325–30. 10.1073/pnas.1424644112 25659742 PMC4345601

[23] Xu XY, Liu B, Wu JS, Jiao LC. Link prediction in complex networks via matrix perturbation and decomposition. Scientific Reports, 2017, 7(1). 10.1038/s41598-017-14847-2 PMC5677011 29116210

[24] Wang WJ, Cai F, Jiao PF, P L. A perturbation-based framework for link prediction via non-negative matrix factorization. Scientific Reports, 2016, 6:38938 10.1038/srep38938 27976672 PMC5156920

[25] Ratha Pech, Hao D, Pan LM, Cheng H, Zhou T. Link Prediction via Matrix Completion. Europhysics Letters,2017,117(3). 10.1209/0295-5075/117/38002

[26] Fond T L, Neville J. Randomization tests for distinguishing social influence and homophily effects. In Proceedings of the World Wide Web Conference (WWW). ACM, New York, 2011, 601–610. 10.1145/1772690.1772752

[27] Kumar R, Novak J, Raghavan P, Tomkins A. Structure and evolution of blogspace. Communications of the ACM, 2004, 47 (12): 35–39. 10.1145/1035134.1035162

[28] Kim M, Leskovec J. Modeling social networks with node attributes using the multiplicative attribute graph model. In Proceedings of the 27th Conference on Uncertainty in Artificial Intelligence(UAI),2011. 10.1080/15427951.2012.625257

[29] Kossinets G, Watts DJ. Empirical analysis of an evolving social network. Science, 2006,311(5757): 88–90. 10.1126/science.1116869 16400149

[30] Yin ZJ, Gupta M, Weninger T, Han JW. LINKREC: a unified framework for link recommendation with user attributes and graph structure. International Conference on World Wide Web, WWW 2010:1211–1212. 10.1145/1772690.1772879

[31] Huang ZC, Ye YM, Li XT, Liu F, Chen HJ. Joint Weighted Nonnegative Matrix Factorization for Mining Attributed Graphs. Advances in Knowledge Discovery and Data Mining. 2017:368–380. 10.1007/978-3-319-57454-7_29

[32] Hsu CC, Lai YA, Chen WH, Feng MH, Lin SD. Unsupervised Ranking using Graph Structures and Node Attributes. Tenth ACM International Conference on Web Search and Data Mining. ACM, 2017:771–779. 10.1145/3018661.3018668

[33] Shi SL, Li YP, Wen YM, Xie W. Adding the sentiment attribute of nodes to improve link prediction in social network. International Conference on Fuzzy Systems and Knowledge Discovery. IEEE, 2015:1263–1269. 10.1109/FSKD.2015.7382124

[34] Mallek S, Boukhris I, Elouedi Z, Lefevre E. Evidential Link Prediction in Uncertain Social Networks Based on Node Attributes. Springer press, 2017: 595–601. 10.1007/978-3-319-60042-0_65

[35] Miller KT., Griffiths TL, Jordan MI. Nonparametric latent feature models for link prediction. In Proceedings of the Neural Information Processing Systems Conference (NIPS), 2009. http://173.236.226.255/tom/papers/linkpred.pdf

[36] A. P. Singh and G. J. Gordon. 2008. Relational learning via collective matrix factorization. In Proceedings of the KDD. Proceedings of the 14th ACM SIGKDD International Conference on Knowledge Discovery and Data Mining, Las Vegas, Nevada, USA, August 24–27, 2008. 10.1145/1401890.1401969

[37] Fan XH, Richard Xu YD, Cao LB, S Y. Learning Nonparametric Relational Models by Conjugately Incorporating Node Information in a Network. IEEE Transactions on Cybernetics, 2017, 47(3):589–599. 10.1109/TCYB.2016.2521376 26887024

[38] Yuan GC, Murukannaiah PK, Zhang Z, Singh MP. Exploiting sentiment homophily for link prediction. 8th ACM Conference on Recommender Systems, 2014:17–24. 10.1145/2645710.2645734

[39] Gong NZ, Talwalkar A, Mackey L, Huang L, Richard Shin E C, Stefanov E, et al Joint Link Prediction and Attribute Inference Using a Social-Attribute Network. Acm Transactions on Intelligent Systems & Technology, 2014, 5(2):1–20. 10.1145/2594455

[40] Z Y, Gao KN, Li F, Y G. A New Method for Link Prediction Using Various Features in Social Networks. Web Information System and Application Conference. IEEE, 2015:144–147. 10.1109/WISA.2014.34

[41] Chen BL, Li FF, Chen SB, Hu RL, Chen L. Link prediction based on non-negative matrix factorization[J]. Plos One, 2017, 12(8):e0182968 10.1371/journal.pone.0182968 28854195 PMC5576740

[42] Backstrom L, Leskovec J. Supervised random walks: predicting and recommending links in social networks. ACM International Conference on Web Search and Data Mining. ACM, 2011:635–644. 10.1145/1935826.1935914

[43] Lee DD, Seung HS. Learning the parts of objects by non-negative matrix factorization. Nature.1999, 401(6755): 788–791. 10.1038/44565 10548103

[44] Wang YX, Zhang YJ. Nonnegative Matrix Factorization: A Comprehensive Review. IEEE Transactions on Knowledge & Data Engineering, 2013, 25(6):1336–1353. 10.1109/TKDE.2012.51

[45] Gemulla R, Nijkamp E, Haas PJ, Sismanis Y. Large-scale matrix factorization with distributed stochastic gradient descent. KDD’11, 2011: 69–77. 10.1145/2020408.2020426

[46] Bao Y, Fang H, Zhang J. TopicMF: simultaneously exploiting ratings and reviews for recommendation. Twenty-Eighth AAAI Conference on Artificial Intelligence. 2014:2–8.

[47] Zhang XC, Zong LL, Liu XY. Constrained Clustering With Nonnegative Matrix Factorization. IEEE Transactions on Neural Networks & Learning Systems, 2016, 27(7):1514–1526. 10.1109/TNNLS.2015.2448653 26241978

[48] Yang Q, Dong EM, Xie Z. Link prediction via nonnegative matrix factorization enhanced by blocks information. In: 2014 10th International Conference on Natural Computation (ICNC), IEEE, 2014:823–827. 10.1109/ICNC.2014.6975944

[49] Vasiloglou N, Gray AG, Anderson DV. Non-Negative Matrix Factorization, Convexity and Isometry. Proc. SIAM Data Mining Conf., 2009: 673–684. 10.1137/1.9781611972795.58

[50] Liu FD, Shan Z, Chen YH. Parallel Nonnegative Matrix Factorization with Manifold Regularization. Journal of Electrical and Computer Engineering, 2018:1–10. 10.1155/2018/6270816

[51] Lazega E. The Collegial Phenomenon: The Social Mechanisms of Cooperation Among Peers in a Corporate Law Partnership. Sociologie du Travail, 2006,48(1):88–109. 10.1016/j.soctra.2006.01.001

[52] McAuley J, Leskovec J. Learning to discover social circles in ego networks. NIPS, 2012: 539–547.

[53] Lu Q, Getoor L. Link-based Text Classification. In Proceedings of the IJCAI Workshop on Text Mining and Link Analysis. 2003.

[54] Zachary W. W. An information flow model for conflict and fission in small groups. Journal of Anthropological Research, 1977, 33(4), 452–473. 10.1086/jar.33.4.3629752

[55] Pablo M. Gleiser, Leon Danon. Community structure in jazz. Advances in Complex Systems, 2003, 6(4): 565–573. 10.1142/S0219525903001067

[56] Batagelj,V. & Mrvar, A. Pajek datasets, available at http://vlado.fmf.uni-lj.si/pub/networks/data/default.htm.

[57] Lada A. Adamic, Natalie Glance. The political blogosphere and the 2004 U.S. election: divided they blog. Proceedings of the 3rd International Workshop on Link Discovery, ACM, 2005, 62(1):36–43. 10.1145/1134271.1134277

[58] Watts D.J., Strogatz S.H. Collective Dynamics of “Small-World” Networks. Nature, 1998, 393: 440–442. 10.1038/30918 9623998

[59] Newman M.E.J. Finding community structure in networks using the eigenvectors of matrices. Physical Review E, 2006, 74(3): 036104 10.1103/PhysRevE.74.036104 17025705

[60] Hanely J.A., McNeil B.J. The meaning and use of the area under a receiver operating characteristic (ROC) curve. Radiology, 1982, 143(1):29–36. 10.1148/radiology.143.1.7063747 7063747

[61] Herlocker JL, Konstann JA, Terveen K, Riedl JT. Evaluating collaborative filtering recommender systems. Acm Trans Information Systems, 2004, 22(1):5–53. 10.1145/963770.963772

[62] Tong HH, Papadimitriou S, Sun JM, Yu PS, Faloutsos C. Colibri: fast mining of large static and dynamic graphs. the 14th ACM SIGKDD international conference on Knowledge discovery and data mining, ACM Press, 2008:686–694. 10.1145/1401890.1401973

